# Low serum serotonin is associated with functional decline, mild behavioural impairment and brain atrophy in dementia-free subjects

**DOI:** 10.1093/braincomms/fcaf005

**Published:** 2025-01-09

**Authors:** Ming Ann Sim, Yingqi Liao, Siew Pang Chan, Eugene S J Tan, Cheuk Ni Kan, Joyce R Chong, Yuek Ling Chai, Narayanaswamy Venketasubramanian, Boon Yeow Tan, Saima Hilal, Xin Xu, Christopher L H Chen, Mitchell K P Lai

**Affiliations:** Department of Anaesthesia, National University Health System, S119074 Singapore, Singapore; Department of Pharmacology, Yong Loo Lin School of Medicine, National University of Singapore, S117600 Singapore, Singapore; Memory Aging and Cognition Centre, National University Health System, S117599 Singapore, Singapore; Department of Pharmacology, Yong Loo Lin School of Medicine, National University of Singapore, S117600 Singapore, Singapore; Memory Aging and Cognition Centre, National University Health System, S117599 Singapore, Singapore; National University Heart Centre, S119074 Singapore, Singapore; Cardiovascular Research Institute, National University of Singapore, S117599 Singapore, Singapore; National University Heart Centre, S119074 Singapore, Singapore; Cardiovascular Research Institute, National University of Singapore, S117599 Singapore, Singapore; Department of Pharmacology, Yong Loo Lin School of Medicine, National University of Singapore, S117600 Singapore, Singapore; Memory Aging and Cognition Centre, National University Health System, S117599 Singapore, Singapore; Department of Pharmacology, Yong Loo Lin School of Medicine, National University of Singapore, S117600 Singapore, Singapore; Memory Aging and Cognition Centre, National University Health System, S117599 Singapore, Singapore; Department of Pharmacology, Yong Loo Lin School of Medicine, National University of Singapore, S117600 Singapore, Singapore; Memory Aging and Cognition Centre, National University Health System, S117599 Singapore, Singapore; Raffles Neuroscience Centre, Raffles Hospital, S188770 Singapore, Singapore; St Luke’s Hospital, S659674 Singapore, Singapore; Department of Pharmacology, Yong Loo Lin School of Medicine, National University of Singapore, S117600 Singapore, Singapore; Memory Aging and Cognition Centre, National University Health System, S117599 Singapore, Singapore; Saw Swee Hock School of Public Health, National University of Singapore, S117549 Singapore, Singapore; School of Public Health, Zhejiang University, Hangzhou 310058, People’s Republic of China; Department of Pharmacology, Yong Loo Lin School of Medicine, National University of Singapore, S117600 Singapore, Singapore; Memory Aging and Cognition Centre, National University Health System, S117599 Singapore, Singapore; Department of Pharmacology, Yong Loo Lin School of Medicine, National University of Singapore, S117600 Singapore, Singapore; Memory Aging and Cognition Centre, National University Health System, S117599 Singapore, Singapore

**Keywords:** serotonin, functional decline, mild behavioural impairment, biomarker

## Abstract

Brain serotonin dysregulation is associated with dementia and neuropsychiatric symptomology. However, the prognostic utility of circulating serotonin levels in detecting features of prodromal dementia including functional decline, cognitive impairment, mild behavioural impairment and brain atrophy remains unclear. In this prospective study of memory clinic subjects followed-up for ≤5 years, dementia-free subjects, classified as having no cognitive impairment or cognitive impairment, no dementia at baseline, underwent annual neuropsychological assessments including Montreal Cognitive Assessment, Global Cognition *Z-*scores and Clinical Dementia Rating Scale Global Scores (where a ≥ 0.5 increment from baseline denotes functional decline). Mild behavioural impairment was measured using baseline and annual Neuropsychiatric Inventory assessments, while brain atrophy was evaluated using cortical and medial temporal atrophy scores from baseline MRI scans. Baseline serum serotonin was then associated with the neuropsychological and neuroimaging measures cross-sectionally and longitudinally. Furthermore, associations of serum serotonin with cross-sectional brain atrophy scores were studied. Of the 191 elderly subjects included in the study, 63 (33.0%) had no cognitive impairment while 128 (67.0%) had cognitive impairment, no dementia. Fourteen subjects (9.0%) showed baseline mild behavioural impairment. Compared with the highest tertile, subjects within the lowest tertile of serotonin had greater Cortical Atrophy scores (adjusted odds ratio = 2.54, 95% confidence interval=1.22–5.30, *P* = 0.013). Serotonin levels were not significantly associated with cross-sectional neuropsychological or mild behavioural impairment scores (all *P* > 0.05). Of the 181 subjects with longitudinal cognitive follow-up (median duration 60.0 months), 56 (30.9%) developed functional decline, while incident mild behavioural impairment occurred in 26/119 (21.8%) subjects. Compared with the highest tertile, lower serotonin levels were associated with higher hazards of functional decline (lowest tertile: adjusted hazards ratio = 2.15, 95% confidence interval = 1.04–4.44, *P* = 0.039), and incident mild behavioural impairment (lowest tertile: adjusted hazards ratio = 3.82, 95% confidence interval = 1.13–12.87, *P* = 0.031, middle tertile: adjusted hazards ratio = 3.56, 95% confidence interval =1.05–12.15, *P* = 0.042). The association between the lowest serotonin tertile and functional decline was mediated via its effect on incident mild behavioural impairment (adjusted odds ratio = 3.96, 95% confidence interval = 1.15–13.61, *P* = 0.029). In conclusion, low circulating serotonin may be associated with cortical atrophy at baseline, as well as act as an early prognostic marker for functional decline and mild behavioural impairment in elderly, dementia-free subjects.

## Teaser

Mild behavioural impairments in the elderly may confer higher risks of subsequent cognitive and functional decline. Sim *et al*. found that lower blood serotonin levels are associated with brain atrophy, functional decline and mild behavioural impairments in a group of elderly, non-dementia participants. Blood serotonin may be a useful biomarker for dementia.

## Graphical Abstract

**Graphical Abstract:**
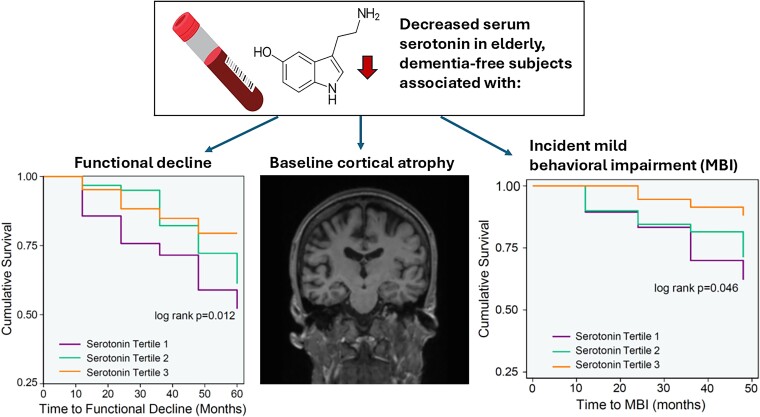

## Introduction

Dementia in old age, the largest proportion of which is associated with Alzheimer’s disease (AD), is an undesirable consequence of aging populations and is characterized by a spectrum of deficits including cognitive decline, functional impairment and neuropsychiatric symptoms (NPS), accompanied by significant patient morbidity, caregiver burden, and healthcare costs associated with institutionalization.^1-3^ Apart from tau and amyloid pathology, it has been increasingly recognized that neurochemical disturbances, such as those involving disturbances of serotoninergic neurotransmission, contribute to pathophysiological processes underpinning cognitive impairment as well as neuropsychiatric behaviours in dementia.^4-7^

Serotonin (5-hydroxytryaptamine, 5-HT) is a monoamine neurotransmitter within the central nervous system, playing vital roles in cognitive and emotive processes.^5,6^ While perturbations of serotoninergic markers like 5-HT transporters and receptors have been correlated with neuropsychiatric as well as cognitive symptoms in mild cognitive impairment and dementia,^6-11^ the potential prognostic roles of 5-HT neurotransmitter in cognitive outcomes, functional decline and mild behavioural impairment (MBI) in pre-dementia subjects remains un-explored. The MBI is a diagnostic construct characterized by persistent behavioural, personality and perception changes, and reflects NPS burden in pre-dementia.^12^ Apart from its significant effects on caregiver stress, MBI has important clinical implications as an at-risk disease entity for cognitive and functional decline.^12,13^ In the setting of cognition, serotonin perturbations have been implicated in the pathogenesis of dementia phenotypes, but these studies have largely been post-mortem, cross-sectional in nature or with limited sample sizes.^4,6,14-20^ Considering the increasingly recognized stage-specific nature of dementia biomarkers, it remains uncertain if earlier findings associated with serotonin dysregulation would be translatable to a cohort of pre-dementia subjects with heterogeneous cognitive disease burden.^21^ Although circulating serotonin levels have been found to correlate with expression within the central nervous system, it is also unclear if the aforementioned associations may yet be detected using measurements of circulating serotonin, in assessing its suitability as a potential blood-based biomarker, an area of active current research.^22^ Given the close associations between serotoninergic function, cognition and behaviours, we hypothesize that lower serotonin levels are associated with neurodegeneration as measured by brain atrophy on neuroimaging, poorer cognitive performance, functional impairment and MBI.

Hence, in this longitudinally assessed, prospective cohort of dementia-free subjects, we evaluated associations of circulating serotonin levels with (i) Baseline neuroimaging markers of brain atrophy; (ii) Baseline cognitive performance, functional impairment and MBI; (iii) Longitudinal cognitive impairment, functional decline and incident MBI. Accordingly, we hypothesized that lower levels of circulating serotonin associated with markers of brain atrophy at baseline, as well as poorer cross-sectional and longitudinal cognitive and functional impairments.

## Materials and methods

### Study participants

This is a longitudinal study involving subjects prospectively recruited from two Singapore-based memory clinics (National University Hospital Singapore and St Luke’s Hospital). Subjects included for this study were recruited from August 2010 to May 2015 and followed-up for a maximum of 5 years with annual neuropsychological and neuropsychiatric assessments by the same assessment team (see below for details of assessments performed). The detailed study protocol has been previously described.^23^ The inclusion criteria were subjects aged ≥50 years, with sufficient language skills to participate in neuropsychological assessments, and who fulfilled a diagnosis of no cognitive impairment (NCI) or cognitive impairment no dementia (CIND). Subjects who fulfilled a diagnosis of major psychiatric conditions listed in Axis I of the Diagnostic and Statistical Manual of Mental Disorders 4th edition (DSM IV), substance abuse disorder, traumatic brain injury resulting in cognitive impairment, tumours, multiple sclerosis and epilepsy were excluded. As we specifically aimed to investigate serum serotonin as an early prognostic marker in pre-dementia, subjects who fulfilled a diagnosis of baseline dementia (based on DSM IV) were also excluded.^24^ Informed written consent was obtained from subjects prior to study recruitment. Institutional Review Board approval was obtained from the National Healthcare Group Domain Specific Review Board (NHG DSRB reference number 2018/01098).

### Assessments of covariates

Data from all subjects regarding sex, age and education levels was collected via detailed questionnaires administered in a standardized fashion. Relevant medical comorbidities such as hypertension, hyperlipidaemia, diabetes and smoking status were collected and verified using medical records. Presence of the apolipoprotein ε4 allele (*APOE4*) was determined using genotyping as previously described.^25^ Subjects with at least one *APOE4* allele were defined as *APOE4* carriers.

### Serum serotonin measurements

Non-fasting blood samples were obtained from study participants at baseline recruitment. Blood samples were drawn into serum separating tubes and centrifuged at 2000g for 10 min at 4°C. Serum fractions were then extracted, aliquoted and stored at −80°C until use. Serum serotonin was measured by competitive enzyme-linked immunosorbent assay (ELISA) according to manufacturer’s instructions (IBL Immuno-Biological Laboratories, Hamburg, Germany). Briefly, serotonin in serum samples and controls were acylated with acetic anhydride in acetone before being applied to 96-well microtiter plates coated with goat anti-rabbit IgG. Serotonin conjugated with biotin and rabbit antiserum were then applied to the plate and incubated overnight on an orbital shaker (500 rpm) at 4°C. The detection system includes the addition of enzyme conjugate (streptavidin alkaline phosphatase) and para-nitrophenyl phosphate. Samples were then read by an ELISA plate reader at 405 nm, and serum serotonin was quantified and analysed using the Biotek Gen5 analysis software (Agilent Technologies, Santa Clara, CA, USA). Intra-assay CV was 3.8–6.6%, and inter-assay CV was 6.7–17.3%.

### Assessments of cognitive performance and functional impairment

Cognitive assessments were performed at baseline, and annually, over a 5-year follow-up period. Assessments performed include the Montreal Cognitive Assessment (MoCA), and a 60-minute locally validated neuropsychological test battery administered by trained research psychologists in the participants’ native language (English, Mandarin or Malay).^26^ This 60-minute neuropsychological battery is based on the Neurological Disorders and Stroke-Canadian Stroke Network (NINDS-CSN) protocol as previously described.^25-27^ Raw scores for all individual tests were then converted to standardized *Z-*scores using the standard deviations (SDs) and mean values of the study reference group of patients with no cognitive impairment. Domain-specific *Z-*scores were then averaged and standardized in a similar fashion, to derive a Global Cognition *Z-*score. On longitudinal follow-up, Global Cognition *Z-*scores were calculated using the means and SDs of the control group in a similar fashion.^27^ Neurocognitive diagnoses of all study participants were discussed at regular consensus meetings attended by study clinicians and neuropsychologists, during which detailed clinical neurocognitive and neuroimaging data were reviewed.^28^ Subjects who were classified as dementia-free at baseline consisted of two subgroups: (i) Those without objective cognitive impairment were categorized as no cognitive impairment (NCI); (ii) Those who displayed impairment in one or more cognitive domains (defined by a score of at least 1.5 SDs below established education-adjusted cut-off values on any component test) but did not exhibit loss of independent daily function were classified as CIND.^29^

To assess functional impairment, clinical dementia rating (CDR) scores were assigned to each patient at baseline, and annually, at every follow-up visit up till 5 years, to stage the severity of cognitive and functional impairment. The CDR rating system is an integrated cognitive and functional assessment, involving detailed standardized interviews conducted by trained research psychologists, evaluating domains including memory, orientation, problem solving and judgement in activities of daily living, personal care, home and hobbies and community affairs as previously described.^28,30^ A global score (CDR-GS) is obtained by evaluating the six domains of function.^31^ The CDR-GS score was employed as a clinically important indicator of functional impairment, due to its validation as a marker of longitudinal function and cognition, aligned with minimal clinically important differences.^31-34^ Therefore, in our study, incident functional decline was defined by a CDR-Global score increment of ≥0.5 at any annual follow-up, as compared with the baseline.

### Assessment of MBI

Ratings for MBI were derived from annual assessments with the Neuropsychiatric Inventory (NPI), with scores based on the frequency and severity of the 10 NPI domains (apathy, depression, anxiety, euphoria, aberrant motor behaviours, agitation, irritability, disinhibition, delusions, hallucinations) being transformed into the five MBI domains (motivation/drive, emotional regulation, impulse control, social inappropriateness and abnormal thoughts/perception) using a published algorithm,^35^ as previously described.^12,36^ Baseline and incident MBI diagnosis were made in accordance with the International Society to Advance Alzheimer’s Research and Treatment of the Alzheimer’s Association (ISTAART-AA) criteria,^37^ whereby baseline MBI was defined as the presence of NPSs (defined as NPI total score of ≥1) over two consecutive years with reference to the baseline. Incident MBI was defined as the development of new NPS over any two consecutive years of follow-up.

### Assessments of neuroimaging markers of brain atrophy

Brain magnetic resonance imaging (MRI) scans were performed at baseline using a 3T Siemens Magnetom Trio Tim Scanner, consisting of a 32-channel head receive coil. Neuroimaging protocols were standardized and included three-dimensional T1 and T2-weighted fluid-attenuated inversion recovery and susceptibility-weighted images. The full brain MRI protocol has been previously described.^26,38^ Semi-quantitative markers of cortical atrophy and medial temporal atrophy on MRI scans were evaluated as markers of brain atrophy as previously described.^11^ Neuroimaging findings were graded an expert rater (S.H.), and gradings by the same observer have previously demonstrated good intra-rater agreement (0.79–0.88).^39^ Cortical atrophy was determined by ventricular, subarachnoid and sulcal dilation on axial sections, and was rated using the 4-point Global Cortical Atrophy scale.^40^ Medial temporal atrophy as determined by widening of choroid fissure, temporal horn and loss of hippocampal height on coronal sections, was graded using the 5-point Scheltens’ scale.^41,42^

### Statistical analysis

Statistical analysis was performed using STATA (StataCorp. 2022. Stata: Release 18) or SPSS software version 29.0 (IBM, Armonk, NY, USA), with *P*-values <0.05 considered statistically significant. Patient characteristics were expressed as percentages for categorical variables and mean ± SD for continuous variables. The χ^2^ tests were used in the analysis of categorical data. Independent Student’s *t*-tests or one-way analyses of variance (ANOVA) tests were used in the analysis of parametric continuous variables. Mann-Whitney U-tests and Kruskal–Wallis tests were used for the analyses of non-parametric data.

In view of its skewed distribution, serum serotonin levels were stratified by tertiles for additional analyses. We employed tests of linear trends (*P*-trend) to assess for significant trends across tertiles, derived from the modelling of serotonin tertiles as numeric variables, in accordance with previously published approaches.^43,44^ Considering its skewed distribution, we have additionally subject serotonin levels to *Z*-score transformation, to approximate a normal distribution, for supplementary analysis as a continuous variable, to better evaluate dose-dependent relationships of the biomarker with the outcomes studied.

Cross-sectional associations of serum serotonin levels with baseline cognitive and neuropsychiatric diagnoses (CIND and MBI) were evaluated using binary logistic regression, with additional adjustments for age, gender, education and *APOE4* status. Cross-sectional analysis to identify associations of serotonin levels with baseline cognition (MoCA and Global Cognition *Z-*scores) and functional impairment (CDR-GS Scores) were performed using linear regression models. The model was adjusted for age, sex, education, *APOE4* status, hypertension, hyperlipidaemia, smoking and diabetes. Cross-sectional associations of serum serotonin levels with cortical atrophy scores and medial temporal atrophy scores on brain MRI were evaluated using ordinal logistic regression. The model was adjusted for age, gender, *APOE4* status and education level. Unadjusted analyses was performed and presented in the supplement.

Longitudinal associations of baseline serotonin levels with functional decline were performed with Cox proportional hazards regression analysis. The model was additionally adjusted for relevant clinical covariates including age, gender, *APOE4* status, years of education, baseline cognition, hypertension, hyperlipidaemia, diabetes and smoking status. Associations between baseline serotonin levels with incident MBI was performed using Cox proportional hazards regression analyses, adjusted for age, gender, *APOE4* status, years of education, baseline cognition, hypertension and hyperlipidaemia. Kaplan-Meier survival curves with log rank tests were additionally constructed for the association of serotonin levels (stratified by tertiles) with functional decline and incident MBI. Longitudinal analysis of associations between baseline serotonin levels with decline in MoCA, and Global Cognition *Z-*scores were performed using Linear Mixed Effects models. The model was adjusted for all relevant baseline cognition, age, gender, *APOE4* status, years of education, hypertension, hyperlipidaemia, diabetes, smoking status and timing of cognitive assessment across longitudinal follow-up. The unadjusted model is presented in the supplement.

As MBI has previously been reported to be associated with functional decline, we sought to investigate the mediation effect of incident MBI on the relationship between baseline serotonin levels and functional decline.^12^ Generalized Structural Equation Modelling (GSEM) analysis with a binomial family distribution and logit link function was employed for mediation analysis between serum serotonin levels, incident MBI, and functional decline. The model could estimate the direct and indirect effects (via incident MBI) of serum serotonin levels on functional decline. The unadjusted model is presented in the supplement.

Sensitivity analysis was performed to include additional adjustments for BMI and to the aforementioned regression models, in view of borderline differences observed between the groups, for presentation within the supplement. We also performed additional statistical adjustments for the use of serotonergic medications (escitalopram (*N* = 1), tramadol (*N* = 1) and mirtazapine (*N* = 4)) within the regression models presented. Furthermore, we performed exploratory analysis to evaluate if cognitive performance differed based on the language of test administration using linear regression of cognitive tests scores, following adjustment for language of test administration, age and years of education.

## Results

### Patient demographics

**Figure 1:**
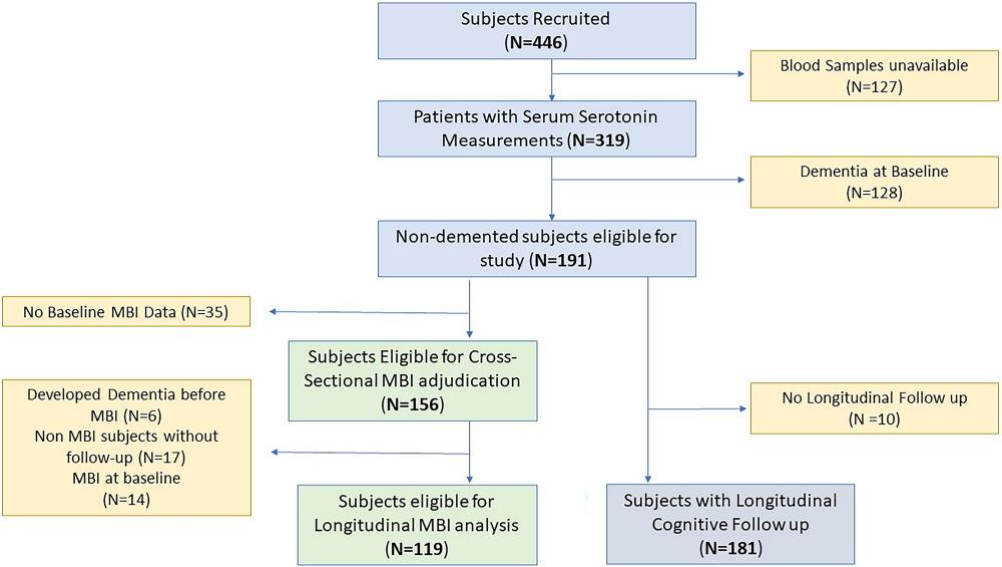
Flowchart of subject inclusion and exclusion.

From 2010 to 2015, 319 of 446 recruited subjects with available blood samples were included. Those with dementia at baseline (*N* = 128) were excluded (Fig. 1—flow chart of subject inclusion and exclusion). Of the remaining 191 dementia-free subjects included for analysis (age 73.4 ± 5.5 years, 48.2% male), 128 (67.0%) had CIND and 63 (33.0%) had NCI. As 35 subjects did not have sufficient NPI data for MBI adjudication at baseline, 14 (9.0%) of the remaining 156 eligible subjects had baseline MBI (Table 1, refer to Fig. 1 for details of subject inclusion and exclusion).

**Table 1:** Subject demographics

Demographic	All subjects (*N* = 191)	Serotonin tertile 1 (*N* = 64)	Serotonin tertile 2 (*N* = 64)	Serotonin tertile 3 (*N* = 63)	*P*-value
Age, years	73.4 ± 5.5	72.9 ± 5.6	74.9 ± 6.0	72.1 ± 4.3	**0**.**010**
Education, years	8.1 ± 5.3	7.8 ± 5.3	7.7 ± 5.3	8.9 ± 5.2	0.361
Gender (male)	92 (48.2)	28 (43.8)	36 (56.3)	28 (44.4)	0.283
Race					
Chinese (%)	156 (81.7)	53 (82.81)	56 (87.5)	47 (74.6)	0.388
Malay (%)	13 (6.8)	5 (7.8)	2 (3.1)	6 (9.5)	
Indian (%)	20 (10.5)	5 (7.8)	5 (7.8)	10 (15.9)	
Others (%)	2 (1.0)	1 (1.6)	1 (1.6)	0 (0.0)	
*APOE4* carrier (%)	47 (24.6)	21 (32.8)	11 (17.2)	15 (23.8)	0.120
BMI (kg/m^2^)	23.9 ± 3.5	24.6 ± 3.7	23.9 ± 3.7	23.2 ± 3.2	0.106
Hypertension (%)	122 (63.9)	43 (67.2)	46 (71.9)	33 (52.3)	0.058
Hyperlipidaemia (%)	142 (74.3)	45 (70.3)	49 (76.6)	48 (76.2)	0.663
Diabetes (%)	58 (30.4)	25 (39.1)	17 (26.6)	16 (25.4)	0.177
Smoking status (%)	46 (24.1)	14 (21.9)	18 (28.1)	14 (22.2)	0.650
Use of serotonergic medications^a^ (%)	6 (3.1)	3 (4.7)	2 (3.1)	1 (1.6)	0.606
Cognitive impairment, no dementia (CIND) (%)	128 (67.0)	45 (70.3)	41 (64.1)	42 (66.7)	0.752
Baseline MBI (%)	14 (9.0)	7 (13.2)	5 (9.6)	2 (3.9)	0.249
MoCA	21.4 ± 4.8	20.4 ± 5.1	21.6 ± 4.7	22.3 ± 4.5	0.069

^a^Of the six subjects receiving concomitant serotonergic medications, four subjects received mirtazapine, one subject received escitalopram and one subject received tramadol. **Bold** font indicates significant *P*-value.

### Cross-sectional associations of serotonin levels with cognitive performance, functional impairment and MBI

Median serum serotonin levels were 95.7 ng/ml, with an interquartile range of 59.4–155.3 ng/ml. Serotonin levels were then stratified by tertiles for subsequent analysis (first tertile: 0–69.98 ng/ml, second tertile: 70.0–132.6 ng/ml and third tertile: 132.6–469.5 ng/ml). Compared with those in the highest tertile, subjects within the lower tertiles of serotonin were older (*P* = 0.010), while other demographics and comorbidities were similar between groups (*P* > 0.05).

The prevalence of cross-sectional CIND (*P* = 0.752), and MBI (*P* = 0.249, Table 1), was not significantly different among the serotonin tertiles. Adjusting for age, gender, education and *APOE4* status, there was no significant association of serotonin levels with CIND or MBI at baseline (all *P* > 0.05, Supplementary Tables S1 and S2, respectively). With respect to neurocognitive testing, the first tertile (compared to third tertile) of serotonin levels was associated with worse MoCA scores (β-1.94, 95% CI −3.60, −0.28 *P* = 0.023) but was attenuated after multivariable adjustment (β-1.23, 95% CI −2.62, 0.17, *P* = 0.084). Serotonin levels were not associated with global cognition *Z-*scores and CDR-Global Scores at baseline on both adjusted and unadjusted analyses (adjusted and unadjusted analyses presented in Supplementary Table S3).

### Cross-sectional associations of serotonin levels with brain atrophy

Ordinal logistic regression of serotonin levels with cortical atrophy scores and medial temporal atrophy (MTA) scores, adjusted for age, gender, APOE4 status and years of education are presented in Table 2. When compared with the highest tertile, subjects within the lowest tertile of serotonin had higher odds of greater cortical atrophy scores (adjusted odds ratio (AOR) 2.54, 95% CI 1.22–5.30, *P* = 0.013, *P*-trend = 0.012, Table 2) measured at baseline. When analysed as a continuous variable, higher serotonin levels similarly associated with lower odds of having greater cortical atrophy scores (AOR 0.63, 95% CI 0.46–0.88, *P* = 0.006). There was no significant association observed between serotonin levels and MTA scores (*P* > 0.05, Table 2). Results of unadjusted analyses are presented in Supplementary Table S4, and are consistent with these findings.

**Table 2:** Association of serotonin levels with neurodegeneration MRI markers at baseline (*N* = 191)

Serotonin tertile	Cortical atrophy score^b^			Medial temporal atrophy score^b^		
	AOR	95% CI	*P*-value	AOR	95% CI	*P*-value
Continuous variable	0.63	0.46–0.88	**0**.**006**	0.87	0.64–1.18	0.366
Stratified by tertiles^a^						
Tertile 1	2.54	1.22–5.30	**0**.**013**	1.50	0.73–0.10	0.271
Tertile 2	1.14	0.54–2.41	0.734	0.82	0.39–1.72	0.606
Tertile 3	ref	ref	ref	ref	ref	ref
*P*-trend	***P* = 0.012**			*P* = 0.267		

^a^Relative to highest tertile (Tertile 3).

^b^Ordinal logistic regression adjusted for age, gender, *APOE4* status and years of education. Regression coefficients expressed as AOR. Serotonin levels were modelled both as continuous terms (z-score transformed to approximate normal distribution), and stratified by tertiles. Tests for linear trends (*P*-trend) were obtained by modelling Serotonin tertiles as numeric variables. **Bold** font indicates significant *P*-value.

### Longitudinal associations of baseline serotonin levels with functional decline and incident MBI

181 subjects had longitudinal cognitive follow up, with 119 subjects being eligible for longitudinal MBI analysis following our previously published approach (Fig. 1).^12^ Over a mean follow up period of 52.97 ± 14.75 months, with a median follow-up of 60 months, 139/181 subjects had completed 5 years follow-up. Of the 181 subjects included for longitudinal analysis 56 subjects (30.9%) developed functional decline, defined as a CDR-GS increment of ≥0.5 from the baseline. Subjects in the first tertile of serotonin (compared to third tertile) were at higher hazard of functional decline, remaining independently associated with 2-times higher hazard on multivariable adjustment (adjusted hazards ratio (AHR) 2.15, 95% CI 1.04–4.44, *P* = 0.039, *P*-trend = 0.035, Table 3). When analysed as a continuous variable, higher serotonin levels similarly associated with lower hazard of functional decline following multivariable adjustment (AHR 0.67, 95% CI 0.46–0.95, *P* = 0.027, Table 3). Unadjusted analyses yielded similar inferences as our adjusted model, and are presented in Supplementary Table S5.

**Table 3:** Association of baseline serotonin levels with functional decline^b^

Serotonin	Decliners/*N* (%) (*N* = 56)	Non-decliners/*N* (%) (*N* = 125)	AHR	95% CI	*P*-value
Continuous variable	56/181 (30.9)	125/181 (69.1)	0.67	0.46–0.95	**0**.**027**
Stratified by tertiles^a^					
Tertile 1	25/58 (43.1%)	33/58 (56.9%)	2.15	1.04–4.44	**0**.**039**
Tertile 2	19/61 (31.1%)	42/61 (68.9%)	1.38	0.64–2.97	0.409
Tertile 3	12/62 (19.4%)	50/62 (80.6%)	Ref	Ref	Ref
*P*-trend			***P* = 0.035**		

^a^With reference to third (highest) tertile.

^b^Cox proportional hazards regression analysis adjusted for age, gender, *APOE4* status, years of education, baseline MoCA scores, hypertension, hyperlipidaemia, diabetes and smoking status. Regression coefficients are expressed as AHR. Serotonin levels were modelled both as continuous terms (*z*-score transformed to approximate normal distribution), and stratified by tertiles. Tests for linear trends (*P*-trend) were obtained by modelling Serotonin tertiles as numeric variables. **Bold** font indicates significant *P*-value.

**Figure 2:**
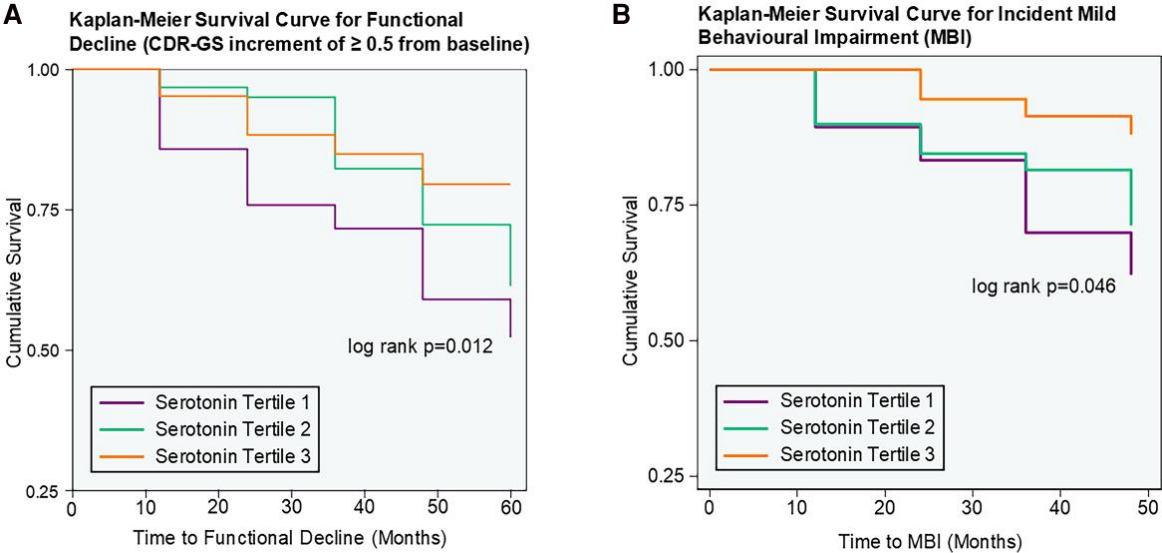
**Longitudinal associations of serum serotonin with functional decline and incident MBI.** Kaplan-Meier curves for serotonin levels (stratified by tertiles) with (**A**) functional decline (as defined by Clinical Dementia Rating—Global Score (CDR-GS) increments of ≥ 0.5 from baseline) and (**B**) incident MBI. *P*-values were derived from log rank tests of serotonin expressed as tertiles, with incident functional decline or MBI, respectively.

Incident MBI was detected in 26/119 (21.8%) subjects (Table 4). When compared with the highest tertile, subjects within the lower two tertiles were both at 3-times higher hazards of incident MBI in multivariable analyses (first tertile: AHR 3.82, 95% CI 1.13–12.87, *P* = 0.031, second tertile: AHR 3.56, 95% CI 1.05–12.15, *P* = 0.042, *P*-trend = 0.035). When analysed as a continuous variable, higher baseline serotonin similarly associated with decreased hazard of incident MBI (AHR 0.53, 95% CI 0.29–0.93, *P* = 0.030). Unadjusted associations of baseline serotonin with incident MBI are presented in Supplementary Table S6 and yielded similar inferences. Kaplan-Meier survival curves for functional decline and incident MBI are presented in Fig. 2A and B.

**Table 4:** Association of baseline serotonin with incident MBI^b^

Serotonin	Incident MBI/*N* (%) (*N* = 26)	Stable/*N* (%) (*N* = 93)	AHR	95% CI	*P*-value
Continuous variable	26/119 (21.8)	93/119 (78.2)	0.53	0.29–0.94	**0**.**030**
Stratified by tertiles^a^					
Tertile 1	12/40 (30%)	28/40 (70%)	3.82	1.13–12.87	**0**.**031**
Tertile 2	10/38 (26.3%)	28/38 (73.7%)	3.56	1.05–12.15	**0**.**042**
Tertile 3	4/41 (9.76%)	37/41 (90.2%)	Ref	ref	Ref
*P*-trend		***P* = 0.035**			

^a^With reference to third (highest) tertile.

^b^Cox proportional hazards regression analysis adjusted for age, gender, *APOE4* status, years of education, baseline MoCA scores, hypertension, hyperlipidaemia, diabetes and smoking status. Regression coefficients expressed as AHR. Serotonin levels were modelled both as continuous terms (*z*-score transformed to approximate normal distribution), and stratified by tertiles. Tests for linear trends (*P*-trend) were obtained by modelling Serotonin tertiles as numeric variables. **Bold** font indicates significant *P*-value.

### Longitudinal associations of baseline serotonin levels with cognitive performance

Associations of baseline serotonin levels by tertiles with longitudinal MoCA, and Global Cognition *Z-*scores are presented in Supplementary Table S7. After adjusting for all relevant baseline cognition, age, gender, *APOE4* status, years of education, hypertension, hyperlipidaemia, diabetes, smoking status and year of cognitive assessment, serotonin levels were not associated with longitudinal decline in MoCA, or global cognition *Z-*scores (all *P* > 0.05, Supplementary Table S7).

### Mediation analysis of baseline serotonin levels with incident MBI and functional decline

**Figure 3:**
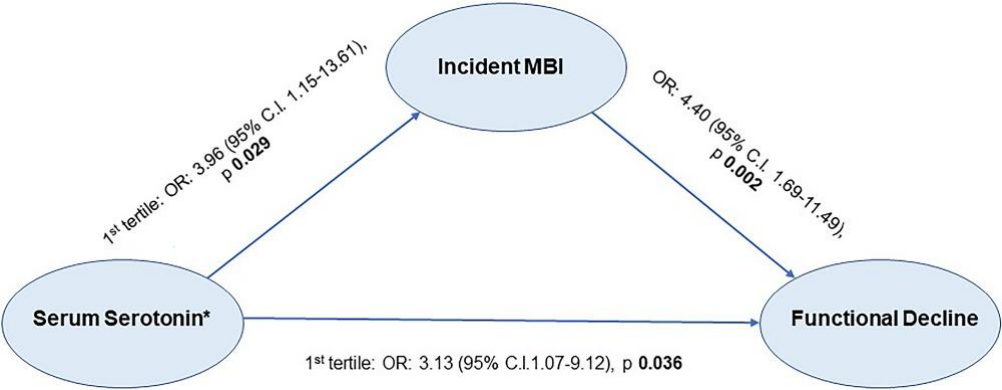
**Mediation analysis of tertile-stratified baseline serotonin levels with functional decline and incident MBI.** *Mediation analyses of incident MBI and functional decline, with serum serotonin levels stratified by tertiles: The analysis was facilitated with GSEM with a binomial family distribution and logit link function. The presented OR refer to the comparison between the first (lowest) and third (highest, and reference) tertile of serum serotonin.

Incident MBI was found to mediate the association between lower serotonin levels and functional decline (Fig. 3). Using GSEM, the lowest tertile of serotonin had a significant direct effect on functional decline (first tertile: Odds ratio [OR]: 3.13, 95% CI 1.07–9.12, *P* = 0.036), when compared with the highest tertile, and additionally, a significant indirect effect on functional decline, mediated via incident MBI (first tertile: OR: 3.96, 95% CI 1.15–13.61, *P* = 0.029). The indirect effect of serum serotonin (for tertile 1 with reference to the tertile 3 (highest tertile)) on functional decline via incident MBI was 7.71 (95% CI: 2.05–28.89).

### Sensitivity analysis

Sensitivity analyses were performed with account for the use of concomitant serotonergic medications in the six patients identified in Table 1. The results of this sensitivity analysis were presented in Supplemental Tables S8–S10. In view of borderline differences in BMI observed between groups, a second set of sensitivity analysis was performed to incorporate additional adjustments for BMI within the analysis. Findings for this analysis are presented in Supplemental Tables S11–S13. Lastly, we performed exploratory analysis to evaluate if cognitive performance differed based on the language of test administration. Language of test administration did not significantly associate with differences in performances on MoCA, and CDR-GS scores (all *P* > 0.05). Details of these analyses are presented in Supplementary Table S14. In summary, sensitivity analyses undertaken revealed inferences, which remained unchanged from our primary analyses findings.

## Discussion

In this study of dementia-free subjects, lower serotonin levels were associated with greater cortical atrophy scores on brain MRI, as well as 3-times higher hazard of functional decline and incident MBI. Furthermore, mediation analyses showed that association of lower serotonin levels with functional decline was mediated through its significant effect on incident MBI, which is in line with established roles of serotoninergic perturbations in NPS. In contrast, serotonin levels were not associated with cognitive function both at baseline and longitudinally. These findings suggest that amongst dementia-free subjects, serotonin may be a useful prognostic biomarker of incident MBI and functional decline, but not of mild cognitive impairment.

Previous studies investigating associations of circulating serotonin with cognitive outcomes are limited and involved either post-mortem or cross-sectional designs. Indeed, lower levels of serotonin and its transporter in dementia have been observed in multiple post-mortem brain studies.^6,8,17^ Several studies also reported decreased platelet or CSF serotonin and its metabolites in AD compared with controls, but these studies used either a cross-sectional design or had relatively small sample sizes.^4,14-16^ In contrast, another study reported higher circulating serotonin levels in vascular dementia, but was not able to completely adjust for cardiovascular risk factors relevant to vascular dementia pathogenesis.^18,45^ A large metabolic phenotyping study from the AddNeuroMed group similarly demonstrated reduced serum serotonin in AD.^46^ However, in view of the cross-sectional nature of these studies, the prognostic role of serotonin in longitudinal outcomes remained unclear. Furthermore, as dementia biomarkers are increasingly recognized to be stage-specific, it remains uncertain if these findings in dementia patients would be translatable to dementia-free patients with considerably less cognitive disease burden, but who would otherwise benefit from disease modifying treatment.^47^ Additionally, while serotonin deficits are known to be associated with mood, behavioural and psychiatric disorders, its prognostic association with NPS burden in the pre-dementia stage remains unevaluated.^5,6^ Considering that serotonin levels within the central nervous system (cerebrospinal fluid) has previously shown good correlations with that measured in the circulation, this further highlights the need for prognostic evaluations of peripherally accessible measures of serotonin, with neuropsychiatric symptomatology and functional decline.^22^

In this study, we showed for the first time the associations of lower circulating serotonin with greater cortical brain atrophy, functional decline and incident MBI. The higher cortical atrophy scores with lower serotonin levels observed in our study are consistent with *in vitro* models demonstrating the protective role of serotonin in neuronal growth and proliferation.^48,49^ Our findings also corroborate molecular imaging studies reporting reduced abundance of brain cortical serotonin transporter levels in cognitively impaired patients, compared with normal controls.^11,50^ However, we did not find an association of serotonin levels with medial temporal atrophy. This could be attributed to several factors. First, this may be due to medial temporal atrophy being preferentially affected in AD pathology, as compared with generalized cortical atrophy,^11,51-53^ or a more direct involvement of serotoninergic alterations in behavioural perturbations. Second, we had employed the Schelten’s visual rating scales in the assessment of medial temporal atrophy due to its clinically intuitive nature, lack of requirements for specialized computational resources, and previously reported good correlations with automated volumetric measurements.^54^ However, it is plausible that the lack of associations with serotonin with medial temporal atrophy could be explained by the relatively lower sensitivity of visual rating scales used in the assessment of changes in hippocampal volume, as compared with automated volumetric methods.^55^

Notably, while lower serotonin levels were associated with greater functional decline on CDR assessment, no significant associations of serotonin levels with cross-sectional or longitudinal cognitive function were found. This lack of association may be attributed to several factors. First, considering that age-related cognitive decline may span decades, it is possible that a longer duration of follow-up may be required to fully elucidate cognitive trajectory in patients with milder cognitive disease burden.^56^ Second, while MoCA and neurocognitive domain-specific tests are objective markers of cognitive performance, they may not capture the spectrum of functional impairment incorporated within the CDR. In contrast MBI captures the NPS burden in the pre-dementia stage—a factor known to correlate with the CDR independently of substantial cognitive deficits discernible on cognitive screening tests.^57,58^ It is therefore plausible that low serotonin levels relate to dementia symptoms and functional impairment via incident MBI, rather than purely cognitive deficits. Indeed, while circulating serotonin is known to correlate with neurobehavioural symptomology, it is postulated that in pre-dementia, neurocognitive function may instead, relate to specific alterations in serotoninergic receptors.^5,11,50,59,60^ This interpretation is supported by the findings of our mediation analysis; demonstrating that incident MBI mediated the association between lower serotonin levels and greater functional decline. This suggests that MBI and objective cognitive performance may represent separate phenomena in the disease process, and despite their correlation, may be associated with differing biological mechanisms. These findings are unsurprising, given that serotonin dysregulations have been associated with a plethora of affective and neurobehavioural symptoms, coupled with the recognition of MBI as an at-risk disease entity for accelerated functional decline.^5,12,59,60^

The observed association of lower serotonin levels with cortical atrophy, functional decline and incident MBI may be explained by several factors. Serotonin is known to play an integral role in the preservation of neurotransmitter integrity, neuromodulation, neuroplasticity, synaptogenesis and neuronal proliferation.^5,6,48,61^ Accordingly, serotoninergic dysfunction within the central nervous system is known to underpin multiple neurological disease states including affective disorders, schizophrenia, as well as behavioural and psychological symptoms of dementia.^5,6,9,59,60,62,63^ The cross-sectional nature of previous studies evaluating associations of serotonin with dementia, raises uncertainties as to whether the observed decrease in serotonin was a consequence of, or rather, an underlying driver of the pathophysiological processes underpinning neurocognitive decline. Our longitudinal study is thus well-poised to address this knowledge gap by establishing the temporality of these associations: demonstrating that lower serotonin levels precede and may thus be a prognostic biomarker for functional decline and MBI. Taken together, higher serotonin levels may confer a protective effect for neuronal proliferation, survival and neurotransmitter integrity, which may collectively contribute to its prognostic role in functional decline and incident MBI.^5,6,48,61^ However, evidence regarding the efficacy of drugs modulating serotonin levels such as selective serotonin reuptake inhibitors in mitigating dementia progression has been inconclusive, further limited by study heterogeneity and small sample sizes.^64^ Our findings therefore extend current knowledge in providing novel evidence of significant associations between lower circulating serotonin levels with cortical atrophy, functional decline and incident MBI, in dementia-free subjects. This highlights the need for future studies and clinical trials to elucidate the therapeutic role for serotonin in modifying the trajectory of neuropsychiatric symptomatology, and functional decline in pre-dementia subjects.

### Study limitations

Our study has several limitations. First, serotonin measurements in this study were performed with ELISA rather than high performance liquid chromatography (HPLC), which is the current gold-standard. However, the ELISA kit utilised within our study has been previously validated to have strong correlations with HPLC data.^65-68^ Second, serotonin concentrations were measured from serum, and levels within the central nervous system (i.e. cerebrospinal fluid, or brain post-mortem tissue sampling) were not studied, which could have affected conclusions drawn. However, circulating serotonin levels have previously been reported to correlate with cerebrospinal fluid levels.^22^ Hence, it may be reasonable to suggest that the circulating levels of serotonin measured within our study are at least partially reflective of serotonin expression in the central nervous system. We had also employed the use of semi-quantitative visual rating scales of cortical and medial temporal atrophy, as opposed to quantitative volumetric analysis programmes, and may thus be less precise than automated methods. Serum serotonin levels were also quantified at baseline, but not repeated at subsequent time points. Therefore, while our study sheds novel insight into the utility of baseline serum serotonin levels for future cognitive and functional outcomes, further studies evaluating the temporal changes in circulating serotonin and their links with cognition and function should be undertaken. The use of serotonergic or psychotropic medications may also affect conclusions drawn between circulating serotonin levels with cognitive and functional outcomes. Although we have aimed to address this through the exclusion of study subjects with major psychiatric illnesses, and adjusted for the use of serotonergic medications within our sensitivity analyses, which revealed unchanged inferences from our primary analysis, these limitations should be considered nonetheless. Additionally, although we found a trend towards lower serotonin levels with higher prevalence of baseline MBI, this did not reach statistical significance. This may be contributed by the relatively small number of patients with MBI at baseline which could have affected statistical power. As our study had been conceived prior to the development of the MBI checklist, the operationalization of two consecutive annual NPI assessments in the ascertainment of MBI was required, in order to fulfil the criterion of persistent NPSs.^37^ This approach may have contributed to a lower rate of MBI, as the breadth of MBI symptomatology outlined in the MBI checklist may not have been fully captured. Nonetheless, the baseline prevalence of MBI in our study remains largely comparable with published rates in current literature.^12,69^ In any case, it is important for our novel findings to be validated in other cohorts.

The availability of serotonin measures at baseline, not at subsequent time points limits the ability to determine how the changes in serotonin over time relate to the changes in cognition and functional outcomes. The use of serotonergic medication or psychotropic drugs at baseline or during the course of follow up would be expected in such a study sample and may affect the NPI and cognitive assessments. This information should be provided for the subjects in the study. The use of peripheral rather than central measures of serotonin could have affected the results as well.

## Conclusions

In subjects without dementia, lower circulating serotonin levels are associated with cortical atrophy, functional decline, and incident MBI. Therefore, serotonin may be a prognostic marker and potential therapeutic target for functional decline and MBI in this group of at-risk subjects.

## Supplementary Material

fcaf005_Supplementary_Data

## Data Availability

The data supporting the findings of this study are available from the corresponding authors, upon reasonable request.
