# Supplementary material for: Low serum serotonin is associated with functional decline, mild behavioural impairment and brain atrophy in dementia-free subjects
Source: Brain Commun. 2025 Jan 9;7(1):fcaf005. doi: 10.1093/braincomms/fcaf005 (PMC11733688; doi:10.1093/braincomms/fcaf005)
Supplement: fcaf005_Supplementary_Data [file fcaf005_supplementary_data.pdf]

Sim *et al*, Low serum serotonin is associated with functional decline, mild behavioural impairment and brain atrophy in dementia-free subjects

SUPPLEMENTARY MATERIALS

Supplementary Table 1: Cross-sectional associations of serotonin levels with baseline CIND

Serotonin	Baseline CIND/ N(%) (N=128)	Baseline NCI / N(%) (N=63)	AOR	95% C.I.	p-value
**Model 1 (adjusted)**					
Continuous variable	128/191 (67.0)	63/191 (33.0)	1.14	0.82-1.57	0.433
Stratified by tertiles*					
Tertile 1	45/64 (70.3)	19/64 (29.7)	0.97	0.42-2.21	0.934
Tertile 2	41/64 (64.1)	23/64 (35.9)	0.55	0.24-1.26	0.155
Tertile 3	42/63 (66.7)	21/63 (33.3)	ref	ref	ref
p-trend	p=0.894				
**Model 2 (unadjusted)**					
Continuous variable	128/191 (67.0)	63/191 (33.0)	0.98	0.72-1.32	0.886
Stratified by tertiles*					
Tertile 1	45/64 (70.3)	19/64 (29.7)	1.18	0.56, 2.51	0.658
Tertile 2	41/64 (64.1)	23/64 (35.9)	0.89	0.43, 1.85	0.758
Tertile 3	42/63 (66.7)	21/63 (33.3)	Ref	ref	ref
p-trend	p=0.660				

*: relative to highest tertile (tertile 3)

Model 1: Binary logistic regression adjusted for age, gender, years of education, *APOE4* status, and serotonin levels. Regression coefficients expressed as Adjusted odds ratio (AOR). Serotonin levels were modelled both as continuous terms (z-score transformed to approximate normal distribution), and stratified by tertiles. Tests for linear trends (p-trend) were obtained by modelling Serotonin tertiles as numeric variables.

Model 2: Unadjusted binary logistic regression. Regression coefficients expressed as unadjusted odds ratio (OR). Serotonin levels were modelled both as continuous terms (z-score transformed to approximate normal distribution), and stratified by tertiles. Tests for linear trends (p-trend) were obtained by modelling Serotonin tertiles as numeric variables.

Supplementary Table 2: Cross-sectional associations of serotonin levels with baseline MBI

Serotonin tertile*	Baseline MBI / N(%) (N=14)	No Baseline MBI / N(%) (N=142)	AOR	95% C.I.	p-value
**Model 1 (adjusted)**					
Continuous variable	14/158 (8.9)	142/158 (89.9)	0.61	0.29-1.26	0.181
Stratified by tertiles*					
Tertile 1	7/53 (13.2)	46/53 (86.8)	4.23	0.82-21.82	0.085
Tertile 2	5/52 (9.6)	47/52 (90.4)	2.43	0.43-13.68	0.315
Tertile 3	2/51 (3.9)	49/51 (96.1)	ref	ref	ref
p-trend	P=0.074				
**Model 2 (unadjusted)**					
Continuous variable	14/158 (8.9)	142/158 (89.9)	0.65	0.33-1.30	0.222
Stratified by tertiles*					
Tertile 1	7/53 (13.2)	46/53 (86.8)	3.73	0.74-18.88	0.112
Tertile 2	5/52 (9.6)	47/52 (90.4)	2.61	0.48-14.10	0.266
Tertile 3	2/51 (3.9)	49/51 (96.1)	ref	ref	ref
p-trend	p=0.106				

*: relative to highest tertile (tertile 3)

Model 1: Binary logistic regression adjusted for age, gender, years of education, *APOE4* status, and serotonin levels. Regression coefficients expressed as Adjusted odds ratio (AOR). Serotonin levels were modelled both as continuous terms (z-score transformed to approximate normal distribution), and stratified by tertiles. Tests for linear trends (p-trend) were obtained by modelling Serotonin tertiles as numeric variables.

Model 2: Unadjusted binary logistic regression. Regression coefficients expressed as unadjusted odds ratios (OR). Serotonin levels were modelled both as continuous terms (z-score transformed to approximate normal distribution), and stratified by tertiles. Tests for linear trends (p-trend) were obtained by modelling Serotonin tertiles as numeric variables.

Supplementary Table 3: Adjusted and unadjusted associations of serotonin levels with cross sectional cognitive performance

Serotonin tertile*	β Coefficient	95% C.I.	p-value
**Model 1 (unadjusted)**			
**MoCA**			
Tertile 1	-1.94	-3.60, -0.28	**0.023**
Tertile 2	-0.72	-2.39, 0.94	0.393
Tertile 3	ref	ref	ref
p-trend	P=0.022		
Continuous variable	0.87	0.19, 1.55	0.012
**Global Cognition Z score**			
Tertile 1*	-0.39	-1.04, 0.26	0.233
Tertile 2*	-0.11	-0.76, 0.54	0.732
Tertile 3	ref	ref	ref
p-trend	P=0.232		
Continuous variable	0.25	-0.02, 0.51	0.065
**CDR-Global Score**			
Tertile 1*	0.07	-0.01, 0.16	0.093
Tertile 2*	-0.01	-0.09, 0.08	0.799
Tertile 3	ref	ref	ref
p-trend	P=0.092		
Continuous variable	-0.03	-0.06, 0.01	0.136
**Model 2 (adjusted)**			
**MoCA**			
Tertile 1*	-1.23	-2.62, 0.17	0.084
Tertile 2*	0.43	-0.99, 1.84	0.552
Tertile 3	ref	ref	Ref
p-trend	P=0.082		
Continuous variable	0.38	-0.20, 0.95	0.198
**Global Cognition Z score**			
Tertile 1*	-0.03	-0.49, 0.43	0.906
Tertile 2*	0.40	-0.07, 0.87	0.095
Tertile 3	ref	ref	ref
p-trend	P=0.888		
Continuous variable	0.02	-0.17, 0.21	0.835
**CDR-Global Score**			
Tertile 1*	0.06	-0.02, 0.15	0.156
Tertile 2*	-0.03	-0.12, 0.06	0.483
Tertile 3	ref	ref	ref

p-trend	P=0.153		
Continuous variable	-0.02	-0.06, 0.02	0.277

*: relative to highest tertile (tertile 3)

Model 1: Unadjusted linear regression model. Regression coefficients are expressed as Beta coefficients. Serotonin levels were modelled both as continuous terms (z-score transformed to approximate normal distribution), and stratified by tertiles. Tests for linear trends (p-trend) were obtained by modelling Serotonin tertiles as numeric variables.

Model 2: Linear regression adjusted for age and gender, years of education, hypertension, hyperlipidaemia, smoking status, diabetes, *APOE4* status**.** Regression coefficients are expressed as Beta coefficients. Serotonin levels were modelled both as continuous terms (z-score transformed to approximate normal distribution), and stratified by tertiles. Tests for linear trends (p-trend) were obtained by modelling Serotonin tertiles as numeric variables.

Supplementary Table 4: Unadjusted Association of Serotonin levels with Cross-sectional Neurodegeneration MRI Markers (N = 191)

**Serotonin**	**Cortical atrophy score** (1)			**Medial temporal atrophy score** (1)		
	**OR**	**95% C.I.**	**p-value**	**OR**	**95% C.I.**	**p-value**
Continuous variable	0.62	0.46-0.85	**0.002**	0.84	0.63-1.11	0.221
Stratified by tertiles*						
Tertile 1	2.58	1.30-5.12	**0.007**	1.48	0.74-2.98	0.269
Tertile 2	1.94	0.98-3.83	0.056	1.39	0.70-2.77	0.350
Tertile 3	ref	ref	ref	ref	ref	ref
p-trend	p=0.007			p=0.267		

*: relative to highest tertile (tertile 3)

(1): Unadjusted ordinal logistic regression of serotonin levels with cortical or medial temporal atrophy scores. Regression coefficients are expressed as odds ratios (OR). Serotonin levels were modelled both as continuous terms (z-score transformed to approximate normal distribution), and stratified by tertiles. Tests for linear trends (p-trend) were obtained by modelling Serotonin tertiles as numeric variables.

Supplementary Table 5: Unadjusted associations of baseline serotonin levels with functional decline (1)

Serotonin	Decliners / N (%) (N=56)	Non decliners / N (%) (N=125)	HR	95% C.I.	p-value
Continuous variable	56/181 (30.9)	125/181 (69.1)	0.60	0.43-0.85	**0.004**
Stratified by tertiles*					
Tertile 1	25/58 (43.1%)	33/58 (56.9%)	2.64	1.33-5.27	**0.006**
Tertile 2	19/61 (31.1%)	42/61 (68.9%)	1.73	0.84-3.57	0.136
Tertile 3	12/62 (19.4%)	50/62 (80.6%)	ref	ref	ref
p-trend	p=0.005				

*: relative to highest tertile (tertile 3)

(1): Cox Proportional Hazards Regression of serotonin levels, with functional decline. Regression coefficients expressed as hazard ratios (HR). Serotonin levels were modelled both as continuous terms (z-score transformed to approximate normal distribution), and stratified by tertiles. Tests for linear trends (p-trend) were obtained by modelling Serotonin tertiles as numeric variables.

Supplementary Table 6: Unadjusted associations of baseline serotonin with incident MBI (1)

**Serotonin**	**Incident MBI / N (%) (N=26)**	**Stable / N (%) (N=93)**	**HR**	**95% C.I.**	**p-value**
Continuous variable	26/119 (21.8)	93/119 (78.2)	0.56	0.34-0.94	**0.028**
Stratified by tertiles*					
Tertile 1	12/40 (30%)	28/40 (70%)	3.50	1.13-10.87	**0.030**
Tertile 2	10/38 (26.3%)	28/38 (73.7%)	2.86	0.90-9.13	0.075
Tertile 3	4/41 (9.76%)	37/41 (90.2%)	ref	ref	ref
P-trend	**p=0.030**				

*: relative to highest tertile (tertile 3)

(1): Cox Proportional Hazards Regression of serotonin levels, with incident MBI. Regression coefficients expressed as hazard ratios (HR). Serotonin levels were modelled both as continuous terms (z-score transformed to approximate normal distribution), and stratified by tertiles. Tests for linear trends (p-trend) were obtained by modelling Serotonin tertiles as numeric variables.

Supplementary Table 7: Longitudinal Associations of Serotonin Levels with MoCA, and Global Cognition *Z*-scores (basic model and fully adjusted model)

Domain	Serotonin	β Coefficient	95% C.I.	p-value
**Model 1 (basic model)**				
**MoCA**	Continuous variable	-0.04	-0.47, 0.39	0.854
	Stratified by tertiles*			
	Tertile 1	0.24	-0.82, 1.31	0.656
	Tertile 2	0.39	-0.66, 1.44	0.465
	Tertile 3	ref	ref	ref
	p-trend=0.647			
**Global Cognition Z score**	Continuous variable	0.01	-0.14, 0.16	0.905
	Stratified by tertiles*			
	Tertile 1	-0.02	-0.39, 0.34	0.906
	Tertile 2	-0.34	-0.70, 0.02	0.068
	Tertile 3	ref	ref	ref
	p-trend=0.875			
**Model 2 (fully adjusted)**				
**MoCA**	Continuous variable	0.05	-0.37, 0.48	0.801
	Stratified by tertiles*			
	Tertile 1	-0.04	-1.10, 1.02	0.945
	Tertile 2	0.54	-0.52, 1.58	0.316
	Tertile 3	ref	ref	ref
	p-trend=0.972			
**Global Cognition Z score**	Continuous variable	0.03	-0.12, 0.18	0.723
	Stratified by tertiles*			
	Tertile 1	-0.06	-0.43, 0.31	0.741
	Tertile 2	-0.26	-0.63, 0.11	0.165
	Tertile 3	ref	ref	ref
	p-trend=0.725			

*: relative to highest tertile (tertile 3)

Model 1: Linear mixed models with salient adjustments made for all relevant baseline cognition, year of cognitive assessment, and serotonin levels. Regression coefficients expressed as Beta coefficients. Serotonin levels were modelled both as continuous terms (z-score transformed to approximate normal distribution), and stratified by tertiles. Tests for linear trends (p-trend) were obtained by modelling Serotonin tertiles as numeric variables.

Model 2: Linear mixed models adjusted for all relevant baseline cognition, age, gender, *APOE4* status, years of education, hypertension, hyperlipidaemia, diabetes, smoking status, BMI, use of serotonergic medications, and year of cognitive assessment. Regression coefficients expressed as Beta coefficients. Serotonin levels were modelled both as continuous terms (z-score transformed to approximate normal distribution), and stratified by tertiles. Tests for linear trends (p-trend) were obtained by modelling Serotonin tertiles as numeric variables.

Supplementary Table 8: Sensitivity analysis for the association of serotonin levels with neurodegeneration MRI markers at baseline, additionally adjusted for concomitant serotonergic medications (N = 191)

**Serotonin**	**Cortical atrophy score (1)**			**Medial temporal atrophy score (1)**		
	**AOR**	**95% C.I.**	**p-value**	**AOR**	**95% C.I.**	**p-value**
Continuous variable	0.63	0.46-0.88	**0.006**	0.87	0.64-1.17	0.358
Stratified by tertiles*						
Tertile 1	2.58	1.23-5.38	**0.012**	1.53	0.74-3.17	0.248
Tertile 2	1.14	0.54-2.43	0.725	0.83	0.40-1.73	0.616
Tertile 3	ref	ref	ref	ref	ref	ref
p-trend	**P=0.011**			P=0.244		

*: relative to highest tertile (tertile 3)

(1): Ordinal logistic regression adjusted for age, gender, *APOE4* status, serotonergic medications and years of education. Regression coefficients expressed as Adjusted odds ratio (AOR). Serotonin levels were modelled both as continuous terms (z-score transformed to approximate normal distribution), and stratified by tertiles. Tests for linear trends (p-trend) were obtained by modelling Serotonin tertiles as numeric variables.

Supplementary Table 9: Sensitivity analysis for the association of baseline serotonin levels with functional decline, additionally adjusted for concomitant serotonergic medications (1)

Serotonin	Decliners / N (%) (N=56)	Non decliners / N (%) (N=125)	AHR	95% C.I.	p-value
Continuous variable	56/181 (30.9)	125/181 (69.1)	0.67	0.47-0.95	**0.025**
Stratified by tertiles*					
Tertile 1	25/58 (43.1%)	33/58 (56.9%)	2.15	1.04-4.46	**0.040**
Tertile 2	19/61 (31.1%)	42/61 (68.9%)	1.35	0.62-2.90	0.450
Tertile 3	12/62 (19.4%)	50/62 (80.6%)	Ref	Ref	Ref
P-trend	**P=0.035**				

*: with reference to highest tertile (tertile 3)

(1): Cox proportional hazards regression analysis adjusted for age, gender, *APOE4* status, years of education, baseline MoCA scores, hypertension, hyperlipidaemia, diabetes, serotonergic medications, smoking status. Regression coefficients are expressed as Adjusted Hazards Ratio (AHR). Serotonin levels were modelled both as continuous terms (z-score transformed to approximate normal distribution), and stratified by tertiles. Tests for linear trends (p-trend) were obtained by modelling Serotonin tertiles as numeric variables.

Supplementary Table 10: Sensitivity analysis for the association of baseline serotonin with incident MBI, additionally adjusted for concomitant serotonergic medications (1)

Serotonin	Incident MBI / N (%) (N=26)	Stable / N (%) (N=93)	AHR	95% C.I.	p-value
Continuous variable	26/119 (21.8)	93/119 (78.2)	0.51	0.29-0.92	0.025
Stratified by tertiles*					
Tertile 1	12/40 (30%)	28/40 (70%)	4.07	1.19-13.88	0.025
Tertile 2	10/38 (26.3%)	28/38 (73.7%)	3.73	1.08-12.84	0.037
Tertile 3	4/41 (9.76%)	37/41 (90.2%)	Ref	ref	Ref
p-trend	P=0.029				

*: with reference to highest tertile (tertile 3)

(1): Cox proportional hazards regression analysis adjusted for age, gender, *APOE4* status, years of education, baseline MoCA scores, hypertension, hyperlipidaemia, diabetes, serotonergic medications, smoking status. Regression coefficients expressed as Adjusted Hazards Ratio (AHR). Serotonin levels were modelled both as continuous terms (z-score transformed to approximate normal distribution), and stratified by tertiles. Tests for linear trends (p-trend) were obtained by modelling Serotonin tertiles as numeric variables.

Supplementary Table 11: Sensitivity analysis for the association of serotonin levels with neurodegeneration MRI markers at baseline, additionally adjusted for BMI (Max N = 191)

**Serotonin**	**Cortical atrophy score (1)**			**Medial temporal atrophy score (1)**		
	AOR	95% C.I.	p-value	AOR	95% C.I.	p-value
Continuous variable	0.64	0.46-0.89	**0.007**	0.86	0.63-1.17	0.327
Stratified by tertiles*						
Tertile 1	2.33	1.10-4.95	**0.027**	1.48	0.70-3.14	0.307
Tertile 2	1.06	0.50-2.29	0.872	0.86	0.41-1.81	0.688
Tertile 3	ref	ref	ref	ref	ref	ref
p-trend	**P=0.025**			P=0.306		

*: relative to highest tertile (tertile 3)

(1): Ordinal logistic regression adjusted for age, gender, *APOE4* status , diabetes, BMI and years of education. Regression coefficients expressed as Adjusted odds ratio (AOR). Serotonin levels were modelled both as continuous terms (z-score transformed to approximate normal distribution), and stratified by tertiles. Tests for linear trends (p-trend) were obtained by modelling Serotonin tertiles as numeric variables.

Supplementary Table 12: Sensitivity analysis for the association of baseline serotonin levels with functional decline, additionally adjusted for BMI (1)

Serotonin	Decliners / N (%) (N=56)	Non decliners / N (%) (N=125)	AHR	95% C.I.	p-value
Continuous variable	56/181 (30.9)	125/181 (69.1)	0.60	0.41-0.89	0.010
Stratified by tertiles*					
Tertile 1	25/58 (43.1%)	33/58 (56.9%)	2.66	1.25-5.64	0.011
Tertile 2	19/61 (31.1%)	42/61 (68.9%)	1.47	0.66-3.30	0.347
Tertile 3	12/62 (19.4%)	50/62 (80.6%)	Ref	Ref	Ref
P-trend	P=0.009				

*: with reference to highest tertile (tertile 3)

(1): Cox proportional hazards regression analysis adjusted for age, gender, *APOE4* status, years of education, baseline MoCA scores, hypertension, hyperlipidaemia, diabetes, BMI, smoking status. Regression coefficients are expressed as Adjusted Hazards Ratio (AHR). Serotonin levels were modelled both as continuous terms (z-score transformed to approximate normal distribution), and stratified by tertiles. Tests for linear trends (p-trend) were obtained by modelling Serotonin tertiles as numeric variables.

Supplementary Table 13: Sensitivity analysis for the association of baseline serotonin with incident MBI, additionally adjusted for BMI (1)

Serotonin	Incident MBI / N (%) (N=26)	Stable / N (%) (N=93)	AHR	95% C.I.	p-value
Continuous variable	26/119 (21.8)	93/119 (78.2)	0.52	0.29-0.93	0.028
Stratified by tertiles*					
Tertile 1	12/40 (30%)	28/40 (70%)	3.99	1.17-13.67	0.027
Tertile 2	10/38 (26.3%)	28/38 (73.7%)	3.74	1.07-13.04	0.038
Tertile 3	4/41 (9.76%)	37/41 (90.2%)	Ref	ref	Ref
p-trend	P=0.032				

*: with reference to highest tertile (tertile 3)

(1): Cox proportional hazards regression analysis adjusted for age, gender, *APOE4* status, years of education, baseline MoCA scores, hypertension, hyperlipidaemia, diabetes, BMI, smoking status. Regression coefficients expressed as Adjusted Hazards Ratio (AHR). Serotonin levels were modelled both as continuous terms (z-score transformed to approximate normal distribution), and stratified by tertiles. Tests for linear trends (p-trend) were obtained by modelling Serotonin tertiles as numeric variables.

Supplementary Table 14: Sensitivity analysis for the association cognitive test performance and language of test administration

Language of test administration*	Regression coefficient	95% C.I.	p-value
**MoCA scores (1)**			
English	Reference	Reference	Reference
Mandarin	0.42	-0.99,1.82	0.565
Malay	-0.39	-3.13,2.36	0.781
Tamil	5.06	-0.44, 10.55	0.071
**CDR-Global Score (2)**			
English	Reference	Reference	Reference
Mandarin	-0.803	-0.12, 0.06	0.491
Malay	-0.06	-0.23, 0.11	0.488
Tamil	-0.28	0.62, 0.07	0.119

*: with reference to the English language

(1): Regression coefficients and 95% confidence intervals were derived from linear regression analysis of MoCA score adjusted for age, years of education, and language of cognitive test administration (in English (N=81), Mandarin (N=98), Malay (N=10), Tamil (N=2)).

(2): Regression coefficients and 95% confidence intervals were derived from linear regression analysis of CDR global scores adjusted for age, years of education, and language of cognitive test administration (in English (N=81), Mandarin (N=98), Malay (N=10), Tamil (N=2)).
